# The Influence of Lifestyle Factors on Resting Energy Expenditure and Its Role in Cardiometabolic Risk: A Cross-Sectional Study

**DOI:** 10.3390/nu17061044

**Published:** 2025-03-16

**Authors:** Joanna Ostrowska, Dorota Szostak-Węgierek

**Affiliations:** Department of Clinical Dietetics, Faculty of Health Sciences, Medical University of Warsaw, E Ciołka Str. 27, 01-445 Warsaw, Poland; dorota.szostak-wegierek@wum.edu.pl

**Keywords:** resting energy expenditure, indirect calorimetry, body composition, biochemical markers, physical activity, total sleep time, diet, accelerometers, cardiometabolic risk

## Abstract

Objectives: This cross-sectional study aimed to examine the associations between lifestyle factors (diet, physical activity, and sleep) and resting energy expenditure (REE) in a group of 75 healthy adults aged 30–45 years without obesity, and to explore its relationship with body composition parameters and selected biochemical markers that could positively influence cardiometabolic disease prevention. Methods: For this purpose, indirect calorimetry, accelerometers, and bioelectrical impedance analysis (BIA) were used. Results: We found that fat-free mass (FFM) showed the strongest association with REE, along with related metrics such as total body water, body cell mass, and muscle mass (*p* < 0.0001, adj. R^2^ > 0.5). In univariable models, all physical activity intensities were significantly associated with REE, but only moderate physical activity (MPA) remained significant after adjusting for sex and FFM (β = 2.1 ± 1.0, *p* < 0.05, adj. R^2^ = 0.589). Similarly, a positive association between HDL-C and REE persisted after adjustments (β = 4.8 ± 2.3 kcal/d, *p* < 0.05, adj. R^2^ = 0.590). Further analyses confirmed that MPA and HDL-C independently contributed to REE (ΔR^2^ = 0.02, *p* < 0.05; Partial r = 0.233 and 0.236, respectively, both *p* < 0.05), highlighting their role beyond the effects of FFM and sex. Other biochemical and lifestyle factors, including HOMA-IR, insulin levels, triglycerides, and total energy intake, showed positive associations with REE in the crude model. However, these relationships diminished after adjustment, suggesting that their influence is likely mediated by factors such as body composition, body size, and sex. Finally, no significant relationship between sleep and REE was observed in our cohort under naturalistic conditions, possibly due to the alignment of participants’ sleep durations with recommended guidelines. Conclusions: These potential direct links between MPA–REE and REE-HDL may be partially explained by habitual, spontaneous physical activity, which contributes to post-exercise metabolic elevation and may promote adipose tissue browning, potentially resulting in favorable metabolic effects, that support cardiometabolic disease prevention.

## 1. Introduction

Total daily energy expenditure (TEE) consists of three main components: resting energy expenditure (REE), the thermic effect of food, and energy expenditure from physical activity. REE, defined as the minimal metabolic rate necessary to sustain life, includes the energy required for essential organ functions at rest and typically accounts for 60–70% of TEE in most individuals [1,2]. Accurately estimating REE is crucial in both scientific research and clinical practice to tailor nutritional strategies and manage conditions such as obesity, malnutrition, and other chronic diseases [1]. REE assessment is particularly important in weight management, as it is essential for determining an appropriate caloric deficit to optimize body composition and prevent cardiometabolic disorders [3]. Miscalculations in REE can result in either inadequate or excessive energy intake, contributing to metabolic imbalances and ineffective weight management in individuals without diagnosed health conditions and clinical populations [3,4].

Among the various methods to assess REE, indirect calorimetry (IC) is considered the “gold standard” [5]. IC determines energy expenditure by measuring a subject’s oxygen consumption (VO_2_) and carbon dioxide production (VCO_2_). The respiratory quotient (RQ), defined as the ratio of exhaled CO_2_ to consumed O_2_, serves as the basis for interpreting REE results [6]. This method enables precise determination of basal nutritional requirements, providing valuable insights for personalized nutritional planning [2].

The influence of lifestyle factors on REE remains an area of ongoing investigation, with findings varying depending on study populations and methodologies. Research indicates that overeating tends to elevate REE, while caloric restriction typically results in its decline, potentially posing challenges for sustaining weight loss over the long term [7]. The impact of physical activity is less clear, with evidence suggesting that resistance training can elevate REE, while the effects of aerobic exercise remain inconsistent [8]. Similarly, while sleep restriction does not appear to directly modify REE, it may contribute to alterations in energy balance by increasing caloric intake [9]. Given these discrepancies, further research using precise metabolic assessment methods is essential to better understand the relationships between diet, physical activity, and sleep in shaping REE, particularly in well-defined, healthy populations.

According to the latest reports from the World Health Organization (WHO) and the International Diabetes Federation (IDF), cardiometabolic diseases, including cardiovascular diseases, type 2 diabetes, and metabolic syndrome, remain among the leading causes of morbidity and mortality worldwide [10,11]. The WHO reports that cardiovascular diseases account for approximately 17.9 million deaths annually, representing 31% of all global deaths [10]. Meanwhile, the IDF estimates that over 463 million adults worldwide are living with diabetes, with this number expected to rise to 700 million by 2045 [11]. Both reports emphasize that lifestyle factors are the primary risk factors for these diseases [10,11]. As a result, they recommend implementing preventive strategies focused on lifestyle modifications to reduce the global burden of cardiometabolic diseases.

While the existing literature already confirms that diet, physical activity, and sleep impact cardiometabolic health [12,13], our study extends this knowledge by exploring whether these factors may also increase REE—a potential mechanism that could contribute to the prevention of cardiometabolic diseases. Studies have shown that higher REE supports weight control, which is crucial in preventing metabolic diseases. Individuals with higher REE have a lower risk of long-term weight gain, reducing the likelihood of developing type 2 diabetes and metabolic syndrome by enhancing glucose regulation and insulin sensitivity [14,15]. Moreover, this study may provide new insights into additional determinants of resting metabolism in a healthy adult population. In addition to indirect calorimetry, we used accelerometry and bioelectrical impedance analysis (BIA), all of which are advanced techniques that offer an objective and precise approach to data collection, providing a significant advantage over self-reported or estimated methodologies.

Therefore, in this study, we aimed to (1) analyze REE (assessed using indirect calorimetry) in a group of 75 healthy adults aged 30–45 years without obesity, and (2) examine the association between lifestyle factors (diet, physical activity, and sleep) and REE, as well as the relationship between REE, body composition parameters, and selected biochemical markers that could positively influence cardiometabolic disease prevention.

## 2. Materials and Methods

### 2.1. Subjects and Data Collection

This cross-sectional study was conducted at the Medical University of Warsaw and included 75 healthy adults (30 men and 45 women) without obesity, who were recruited through advertisements. The inclusion criteria were age 30–45 years, no diagnosed chronic diseases, and a BMI between 18.5 kg/m^2^ and 29.9 kg/m^2^. Exclusion criteria included pharmacological treatment and contraindications for body composition analysis, such as epilepsy, implanted cardiac pacemakers, defibrillators, or metal endoprostheses.

Body weight and height were measured using a Seca 799 measurement station and column scales with a precision of ±0.1 kg/cm. Waist circumference was measured with a steel measuring tape, positioned midway between the lower border of the ribs and the iliac crest in the horizontal plane. The study protocol was approved by the Ethics Committee of the Medical University of Warsaw (KB/158/2021), and all participants provided written informed consent.

### 2.2. Resting Energy Expenditure Measured by Indirect Calorimetry

REE was measured using indirect calorimetry (Q-NRG+, COSMED Srl, Rome, Italy) in oronasal face mask mode with an external turbine flowmeter. The flowmeter and sampling line were connected to the mask. REE was calculated based on the measurement of oxygen consumption (VO_2_) and carbon dioxide production (VCO_2_), along with other ventilatory parameters. Prior to each test session, the instrument was warmed up and calibrated automatically. To maintain high measurement accuracy, automatic device calibration was performed before each test, with additional monthly two-point gas analyzer calibration and turbine verification using a calibration syringe, following the manufacturer’s guidelines. In clinical evaluations, the system demonstrated CV% ≤ 1% for VO_2_ and EE, and CV% ≤ 1.5% for VCO_2_ and RQ, confirming its consistency and precision across different tests [16,17,18]. All tests were conducted in the morning (08:30–11:00 a.m.) after a 12 h fast and 12 h avoidance of strenuous exercise. Participants were instructed to avoid speaking and to relax without falling asleep during the test. Data were collected over a 20 min interval, with the first 5 min used for familiarization and excluded from analysis. The VO_2_ and VCO_2_ values were used to calculate the respiratory quotient (RQ) as the ratio of VCO_2_/VO_2_, and REE (in kcal/day) was derived using the Weir equation [19]. Participants with RQ values outside the physiological range (0.7–1.0) were excluded from the analysis.

### 2.3. Bioelectrical Impedance Analysis, Biochemical Tests, Physical Activity, and Sleep Duration

The methodology for bioelectrical impedance analysis, biochemical tests, physical activity measurement, and sleep duration assessment were described in detail in our previous publication (Ostrowska et al. [20]). As previously described, body composition was assessed using bioelectrical impedance analysis (Bioscan 920-2, Maltron Int, Rayleigh, Essex, UK), following ESPEN guidelines. Biochemical tests included fasting serum measurements of insulin, glucose, lipid profiles, and high-sensitivity C-reactive protein. The Homeostatic Model Assessment of Insulin Resistance (HOMA-IR) was calculated based on fasting insulin and glucose concentrations. Physical activity and sleep duration were monitored over 7 days using wGT3X-BT ActiGraph accelerometers (ActiGraph LLC, Pensacola, FL, USA), with physical activity intensity classified using Freedson’s cut-offs [21]. The Cole–Kripke algorithm [22] was used to calculate sleep and wake periods. Data from participants with fewer than 4 nights of data were excluded from analyses.

### 2.4. Statistical Analyzes

Descriptive statistics were used to summarize the collected data. Quantitative variables were presented as means and standard deviations after assessing their distribution with the Shapiro–Wilk test. Comparisons of continuous variables between males and females were conducted using the independent samples *t*-test. To evaluate the influence of various factors on REE, univariable linear regression analysis was performed (crude model). These models were subsequently adjusted for sex and fat-free mass (FFM) (adjusted model, Model 1). The adjusted coefficient of determination (adj. R^2^) and *p*-value were used to assess the predictive power of each factor. Prior to conducting the regression analysis in Model 1, the linearity of each relationship was verified.

To assess the independent contribution of biochemical and lifestyle parameters that showed statistical significance in Model 1 to REE beyond sex and FFM, we calculated the change in adjusted R^2^ (ΔR^2^), which quantifies the increase in explained variance after adding each parameter to the regression model. Additionally, we reported the partial correlation coefficient (Partial r) to reflect the strength of the independent association between each parameter and REE after adjusting for sex and FFM.

All analyses were conducted using R statistical software, version 3.4.0 with the statistical significance level set at *p* < 0.05.

## 3. Results

The study included 75 participants (30 men and 45 women) with a mean age of 37 years, average weight of 72 kg, height of 173 cm, and BMI of 24 kg/m^2^. No significant differences in age or percentage of fat-free mass (FFM) were observed between males and females (*p* > 0.05). However, men had significantly higher absolute FFM (63 vs. 44 kg, *p* ≤ 0.001) and a lower average percentage of fat mass (26% vs. 29%, *p* < 0.05) compared to women. Other body composition components also showed significant differences between sexes (*p* < 0.05).

Regarding metabolic markers, no sex-specific differences were found in HOMA-IR, total cholesterol (TC), LDL-C, and CRP levels. However, men had significantly higher triglyceride (TG) levels (119 vs. 79 mg/dL, *p* < 0.001) and lower HDL-C levels (53 vs. 67 mg/dL, *p* < 0.001) compared to women. In terms of lifestyle factors, men engaged in significantly more physical activity (MVPA, including both moderate and vigorous PA) than women (90 vs. 57 min/day, *p* < 0.05). Dietary intake also varied by sex, with men reporting higher energy, protein, and fat intake, while no differences in sleep duration were observed between sexes. Descriptive characteristics of the study population are summarized in Table 1.

### 3.1. Impact of Anthropometric and Body Composition Factors on REE

Table 2 summarizes the influence of anthropometric and body composition parameters on REE. Among the basic parameters, body weight, height, BMI, and waist circumference showed strong associations with REE, with body weight having the highest predictive power in this group (β = 19.8 ± 2.4 kcal/d, *p* < 0.0001, adj. R^2^ = 0.523). Age did not have a significant impact on REE, likely due to the deliberate selection of participants within a similar age range.

In the body composition parameters, measures related to fat-free mass (FFM), including protein mass, muscle mass, dry weight, body cell mass (BCM), and water compartments (total body water [TBW], extracellular water [ECW], and intracellular water [ICW]), demonstrated strong associations with REE, consistent with the high energy demands of metabolically active tissue. Among these, FFM (β = 28.8 ± 2.8 kcal/d, *p* < 0.0001, adj. R^2^ = 0.572) was the most substantial predictor, reinforcing the role of lean mass in determining resting energy requirements.

In contrast, fat mass (FM) showed the lowest predictive power for REE among body composition parameters (*p* < 0.001, adj. R^2^ = 0.171). This effect was especially weak for subcutaneous adipose tissue (SAT), with subcutaneous fat area demonstrating minimal association with REE (*p* < 0.05, adj. R^2^ = 0.064). Visceral adipose tissue (VAT), however, exhibited a slightly stronger association with REE, reflecting its higher metabolic activity compared to SAT.

### 3.2. Impact of Biochemical Factors on REE

Regarding biochemical factors, in the crude model, a significant inverse relationship was found between REE and HDL-C (β = −6.6 ± 3.1 kcal/d, *p* < 0.05, adj. R^2^ = 0.045). Positive significant associations were observed for TG (β = 3.3 ± 0.9 kcal/d, *p* < 0.001, adj. R^2^ = 0.144), HOMA-IR (β = 83.1 ± 36.1 kcal/d, *p* < 0.05, adj. R^2^ = 0.055), and fasting insulin levels (β = 22.1 ± 9.6 kcal/d, *p* < 0.05, adj. R^2^ = 0.054). After adjustment for sex and FFM in Model 1, the significant associations of REE with TG, HOMA-IR, and insulin levels were no longer present. Notably, the association with HDL-C remained significant in Model 1, but the relationship became positive instead of inverse (β = 4.8 ± 2.3 kcal/d, *p* < 0.05, adj. R^2^ = 0.590). These results suggest that the relationship between HDL-C and REE may be more robust, while associations with TG, HOMA-IR, and insulin are likely influenced by body composition and demographic factors (Table 3).

### 3.3. Impact of Lifestyle Factors (Physical Activity, Sleep, Diet) on REE

Table 4 presents linear regression models evaluating lifestyle predictors of REE. All levels of physical activity intensity were significantly associated with REE in the crude model. However, after adjusting for sex, and FFM, the association with VPA was no longer significant. Moderate physical activity (MPA) and moderate-to-vigorous physical activity (MVPA) retained significant associations with REE in the adjusted model (MPA: β = 2.1 ± 1.0 kcal/d, *p* < 0.05, adj. R^2^ = 0.589; MVPA: β = 1.5 ± 0.8 kcal/d, *p* < 0.05, adj. R^2^ = 0.589). Sleep duration showed no significant association with REE in either the crude or adjusted models.

For dietary intake, energy was the only significant predictor of REE in the crude model (β = 0.7 ± 0.2 kcal/d, *p* < 0.01, adj. R^2^ = 0.218). After adjusting for sex and FFM, energy remained the main predictor, though the association approached but did not reach statistical significance (*p* = 0.06). Protein, fats, and carbohydrates showed minimal and non-significant associations with REE in both models (Table 4).

### 3.4. Effects of Physical Activity and HDL-C on REE Beyond FFM and Sex

Table 3 and Table 4 present the associations between biochemical and lifestyle factors and REE, adjusted for FFM and sex. Since only MPA, MVPA, and HDL-C remained significant in these models, their independent contributions to REE were further examined in Table 5.

In this additional analysis, we assessed the change in explained variance (ΔR^2^) after adding each variable to the base model (FFM + sex). The results showed that including MPA improved the model fit (ΔR^2^ = 0.02, *p* < 0.05), confirming its independent association with REE. This was further supported by the partial correlation analysis, which demonstrated a positive relationship between REE and MPA (r = 0.233, *p* < 0.05) after adjusting for FFM and sex.

Similarly, adding MVPA resulted in a ΔR^2^ of 0.02 (*p* < 0.05), indicating its independent contribution to REE. The partial correlation coefficient (r = 0.232, *p* < 0.05) indicates that MVPA contributes to REE to a similar extent as MPA.

Finally, the inclusion of HDL-C also led to improvement in the model (ΔR^2^ = 0.02, *p* < 0.05), with a corresponding partial correlation of r = 0.236 (*p* < 0.05), indicating a modest but statistically significant relationship with REE.

## 4. Discussion

In this cross-sectional study, we aimed to investigate the associations between lifestyle factors and REE. Additionally, we examined the potential role of REE in modulating cardiometabolic risk through its interactions with biochemical and body composition and anthropometric parameters. To the best of our knowledge, no previous studies have comprehensively focused on the anthropometric, biochemical, and lifestyle factors contributing to individual variations in REE among healthy individuals. Moreover, our study employed objective methods to assess body composition (multi-frequency bioelectrical impedance), REE (indirect calorimetry), and physical activity and sleep quality (tri-axial accelerometers), reducing the limitations associated with self-reported data.

### 4.1. Associations Between REE and Body Composition

The results of our study align with findings emphasizing the strongest association of BIA-derived variables with REE [14,23]. FFM, alongside components like total body water, body cell mass (BCM), and muscle mass, demonstrated the highest predictive power for REE. These variables, linked to metabolically active tissues, outperform basic anthropometric parameters such as body weight, height, and BMI [14]. Interestingly, fat mass (FM) showed a significantly weaker association with REE, particularly subcutaneous adipose tissue (SAT), which had minimal predictive value compared to visceral adipose tissue (VAT). This discrepancy is likely due to SAT’s lower metabolic activity and distinctive gene expression patterns, including higher adiponectin and reduced proinflammatory adipokine expression [23,24].

### 4.2. Biochemical Correlates of REE

Another aspect influencing REE is immunological function, which accounts for up to 15% of daily energy expenditure [25]. C-reactive protein (CRP) is commonly used as a biomarker of inflammation [25,26]. Most studies on REE-CRP associations focus on critically ill patients, including those with pancreatic cancer [27], sepsis [28], or chronic kidney disease [29]. However, our study did not find a significant association between REE and CRP in individuals with normal weight and overweight.

Similarly, the initial association between HOMA-IR and REE was no longer significant after adjusting (Table 3, Model 1), supporting the idea that insulin resistance and REE are largely mediated by body size rather than a direct metabolic effect. Increased body mass is associated with oxidative stress, proinflammatory cytokine production, and chronic inflammation, which can contribute to insulin resistance [26,30,31]. A similar trend was observed for TG, where a significant association in crude models disappeared after adjustment.

### 4.3. The Role of Physical Activity and HDL-C in REE

A key finding of our study was the persistent association between MPA and REE, even after adjusting for sex and FFM (Table 4 and Table 5). Although previous research has yielded mixed results regarding the impact of physical activity on REE, some evidence suggests that resistance training may be more effective than aerobic exercise in driving long-term increases in metabolic rate [8,32]. However, our findings highlight that habitual, spontaneous movement (named in our publication as MPA)—occurring more frequently than VPA—may offer metabolic benefits beyond those of high-intensity exercise alone. This effect may be partially explained by compensatory behaviors that vary between different exercise intensities. VPA can lead to increased sedentary behavior post-exercise, which may offset the energy expenditure benefits gained during the exercise session [33,34]. In contrast, this compensatory behavior appears to be less pronounced with MPA. Consequently, in adults, MPA is a key determinant of overall physical activity levels [35]. Furthermore, regular physical activity has been shown to contribute to post-exercise REE elevation [36,37]. In addition, physical activity (including MPA) has been shown to induce adipose tissue browning, thereby enhancing insulin sensitivity and thermogenic activity. These adaptations are associated with increased expression of UCP1 (Uncoupling Protein 1), which plays a key role in thermogenesis and fat oxidation, potentially contributing to a sustained elevation in REE [38,39]. Additionally, regular physical activity is known to increase HDL-C levels [40], which may explain the observed relationship between HDL-C and REE in our adjusted model.

### 4.4. Lack of Association Between Sleep and REE

Efficient sleep duration is increasingly recognized as an important factor in maintaining normal body weight [41]. However, the relationship between sleep and REE remains less well characterized. While some studies suggest that decreased sleep time is associated with an increased RQ and REE [42,43], the majority indicate that sleep restriction does not directly modify REE but may instead contribute to alterations in energy balance by increasing caloric intake [9]. This is consistent with the findings of our study, as we also did not observe a direct association (Table 4). These discrepancies may stem from variations in participant characteristics and study designs–for instance, a cross-sectional study on 126 individuals with obesity who regularly slept less than 6.5 h per night found that poor sleep quality was associated with increased REE, likely due to stress system activation. This study estimated that a 10 μg/dL increase in serum cortisol could elevate REE by approximately 10% [42]. Similarly, in a study of healthy participants with a lean body mass, experimentally induced total sleep deprivation and sleep fragmentation led to an approximate 7% increase in energy expenditure [44]. In contrast, our study was conducted under naturalistic conditions, with participants’ mean total sleep time aligning with the National Sleep Foundation’s recommendations of around 7.5 h per night (Table 1) [45]. These naturalistic conditions likely influenced the outcomes, minimizing the impact of sleep deprivation on REE.

### 4.5. Dietary Intake and REE

Other lifestyle factors, such as diet, also play a role in modulating REE. Research indicates that overeating tends to elevate REE, while caloric restriction typically leads to its decline, which may pose challenges for sustaining long-term weight loss [7]. Moreover, higher protein intake has been associated with increased REE—for example, a 1% increase in protein intake can raise REE by approximately 3 kcal/day [46]. Additionally, high-protein diets are well-documented for their role in preserving fat-free mass (FFM) and maintaining REE during weight loss [47]. However, this relationship was not confirmed in our study. In the crude model, energy intake significantly predicted REE, whereas protein intake demonstrated only a marginal and non-significant effect. After adjustment, the predictive power of energy intake on REE was further reduced, indicating that the impact of energy on REE may be mediated by factors such as sex and changes in body composition.

### 4.6. Limitations of the Study

Some limitations of the present study should be considered when interpreting the findings. While the accelerometers used are reliable for capturing overall activity patterns, they have lower sensitivity to sedentary behaviors and are unable to effectively register static exercises or physical activities that do not involve a transfer of the center of mass, such as carrying a load [48]. Additionally, the female participants were examined without accounting for their menstrual cycle phases, which can influence several physiological parameters, including REE and sleep, potentially introducing minor variability [49]. Furthermore, the relatively small sample size (*n* = 75) limits the generalizability of our findings, and the cross-sectional design does not allow for establishing causality. Although there was an unequal distribution of male and female participants, sex was included as a covariate in the adjusted models (adjusted for sex and FFM), minimizing its potential impact on the observed associations.

## 5. Conclusions

This study provides some new insights into the associations between lifestyle factors, body composition, and REE in a cohort of healthy individuals.

Our findings reinforce the central role of FFM and related body composition metrics (linked to metabolically active tissues) as having the strongest association with REE, surpassing traditional anthropometric measures such as BMI and body weight.

While energy intake demonstrated a significant association with REE in the crude model, this relationship diminished after adjustment, suggesting that its influence is likely mediated by factors such as body composition and sex. Interestingly, associations between protein intake and REE, well-supported in the literature, were not confirmed in this study. Similarly, the relationship between sleep and REE was not demonstrated in our cohort under naturalistic conditions, possibly due to the alignment of participants’ sleep durations with recommended guidelines.

In contrast, our findings revealed a significant potential link between MPA and REE, with particular emphasis on frequently undertaken moderate physical activity and its associated increase in HDL-C concentrations. These potential direct links between MPA–REE and REE-HDL could be explained by habitual, spontaneous physical activity, which occurs more frequently than structured exercise and contributes to post-exercise metabolic elevation. This activity may also promote adipose tissue browning, potentially resulting in favorable metabolic effects that support cardiometabolic disease prevention.

Future research should aim to further explore these relationships in larger, more diverse populations and under controlled conditions to enhance our understanding of the mechanisms driving energy expenditure and its broader implications for cardiometabolic health.

## Figures and Tables

**Table 1:** Characteristics of study participants.

	Total, *n* = 75		Females, *n* = 45		Males, *n* = 30		
Basic Parameters	Mean (SD)	Range	Mean (SD)	Range	Mean (SD)	Range	*p*-Value
Age (years)	37 (5.0)	28.0, 46.0	36 (4.5)	28.0, 45.0	38 (5.1)	30.0, 45.0	ns.
Body weight (kg)	72 (14.0)	44.0, 105.0	63 (8.3)	44.0, 82.0	85 (10.5)	57.0, 107.0	<0.001
Height (cm)	173 (10.0)	150.0, 194.0	167 (6.5)	150.0, 178.0	181 (6.0)	171.0, 194.0	<0.001
BMI (kg/m^2^)	24 (3.1)	18.6, 29.5	23 (2.5)	18.6, 28.1	26 (2.9)	18.6, 29.5	<0.001
WC (cm)	84 (11.5)	63.0, 110.0	78 (8.1)	63.0, 91.0	93 (9.6)	65.0, 110.0	<0.001
**Body composition parameters**							
FFM (kg)	52 (10.1)	34.3, 72.0	44 (3.7)	34.3, 51.2	63 (5.5)	46.2, 72.0	<0.001
FFM (%)	72 (5.6)	0.7, 84.3	71 (5.7)	60.8, 83.6	72 (14.0)	0.7, 84.3	ns.
FM (kg)	20 (6.5)	8.8, 42.2	19 (5.6)	9.7, 32.1	23 (6.9)	8.8, 42.2	<0.01
FM (%)	28 (5.5)	15.7, 39.2	29 (5.5)	19.8, 39.2	26 (5.1)	15.7, 38.4	<0.05
VAT (cm^2^)	118 (83.0)	21.0, 350.0	84 (50.2)	30.0, 276.0	171 (92.7)	21.0, 350.0	<0.001
SAT (cm^2^)	98 (35.0)	28.0, 201.0	88 (32.3)	28.0, 201.0	110 (35.5)	46.0, 173.0	<0.05
VAT/SAT	1 (0.6)	0.3, 2.9	1 (0.3)	0.3, 2.0	2 (0.7)	0.5, 2.9	<0.001
TBW (Lt)	37 (7.8)	24.0, 54.5	31 (2.9)	24.0, 36.9	46 (4.1)	34.6, 54.5	<0.001
TBW (%)	51 (3.6)	41.9, 62.9	50 (3.1)	41.9, 57.0	53 (3.4)	46.6, 62.9	<0.001
ECW (Lt)	17 (2.7)	12.2, 22.8	15 (1.5)	12.2, 19.3	19 (1.7)	14.9, 22.8	<0.001
ECW (%)	45 (7.5)	0.4, 69.8	48 (6.4)	15.5, 69.8	41 (7.6)	0.4, 46.6	<0.001
ICW (L)	21 (6.2)	7.3, 47.7	17 (5.1)	7.3, 47.7	26 (2.6)	19.2, 31.9	<0.001
ICW (%)	54 (4.4)	30.2, 59.0	52 (4.3)	30.2, 56.2	57 (1.2)	53.4, 59.0	<0.001
ECW/ICW	1 (0.2)	0.7, 2.3	1 (0.2)	0.8, 2.3	1 (0.0)	0.7, 0.9	<0.001
BCM (kg)	27 (6.2)	14.1, 41.1	23 (2.3)	14.1, 26.9	34 (3.1)	25.1, 41.1	<0.001
ECM (kg)	25 (4.3)	17.4, 34.6	22 (1.8)	17.4, 25.9	29 (2.8)	21.1, 34.6	<0.001
Protein mass (kg)	11 (2.4)	6.6, 18.2	9 (1.1)	6.6, 11.5	13 (2.1)	8.6, 18.2	<0.001
Muscle mass (kg)	24 (6.5)	12.6, 37.8	19 (1.8)	12.6, 22.6	31 (3.2)	22.8, 37.8	<0.001
Dry weight (kg)	70 (14.7)	38.2, 108.3	61 (8.7)	38.2, 78.7	84 (10.7)	53.3, 108.3	<0.001
**Biochemical parameters**							
TC (mg/dL)	199 (30.2)	107.3, 268.8	199 (26.9)	150.9, 262.4	200 (34.5)	107.3, 268.8	ns.
HDL-C (mg/dL)	62 (14.6)	35.2, 105.0	67 (14.4)	44.2, 105.0	53 (9.5)	35.2, 76.0	<0.001
LDL-C (mg/dL)	120 (24.0)	62.0, 190.0	116 (21.6)	62.0, 170.0	126 (26.4)	93.0, 190.0	ns.
TG (mg/dL)	95 (48.1)	35.3, 340.0	79 (26.4)	38.2, 144.9	119 (60.5)	35.3, 340.0	<0.001
Fasting blood glucose (mg/dL)	97 (7.3)	80.0, 118.0	97 (5.9)	82.0, 111.0	99 (8.9)	80.0, 118.0	ns.
Fasting insulin (μU/mL)	8 (4.6)	2.2, 25.0	7 (2.9)	2.8, 14.2	10 (5.9)	2.2, 25.0	ns.
HOMA-IR	2 (1.2)	0.5, 6.4	2 (0.7)	0.6, 3.4	2.4 (1.6)	0.5, 6.4	ns.
CRP (mg/L)	1 (2.8)	0, 2.4	1 (2.0)	0, 5.8	2 (4.2)	0, 2.4	ns.
**Indirect calorimetry parameters**							
VO_2_ (ml/min)	249 (54.6)	156.8, 370.7	219 (35.3)	155.8, 297.4	294 (46.0)	191.9, 370.7	<0.001
VCO_2_ (ml/min)	227 (55.0)	135.4, 371.1	198 (34.3)	135.4, 293.1	271 (50.5)	189.6, 371.1	<0.001
RQ factor	0.91 (0.06)	0.8, 1.0	0.91 (0.05)	0.81, 1.0	0.92 (0.06)	0.78, 1.0	ns.
REE (kcal/day)	1761 (397.0)	1089.7, 2823.6	1543 (247.4)	1089.7, 2120.8	2088 (345.5)	1384.4, 2823.6	<0.001
**Physical activity and sleep parameters**							
MPA (min/day)	61 (31.6)	22.8, 183.0	53 (17.0)	22.8, 100.1	74 (42.4)	30.3, 183.0	<0.05
VPA (min/day)	9 (15.5)	0.0, 60.0	5 (7.6)	0.1, 31.6	15 (21.2)	0.0, 60.8	<0.05
MVPA (min/day)	70 (44.2)	23.6, 236.5	57 (20.0)	23.6, 114.5	90 (61.2)	34.3, 236.5	<0.05
TST (min/night)	455 (58.2)	289.0, 609.0	458 (69.0)	289.0, 609.0	451 (35.8)	387.1, 518.2	ns.
**Diet parameters**							
Energy (kcal/d)	2051 (449.3)	1287.0, 3132.4	1801.0 (265.5)	1287.0, 2526.3	2449.3 (391.9)	1653.6, 3132.4	<0.001
Protein (g/d)	85.6 (24.2)	28.0, 137.2	73.2 (16.7)	28.0, 102.3	105 (21.30)	76.7, 137.2	<0.001
Fats (g/d)	78 (21.3)	43.8, 150.8	71 (15.2)	43.8, 108.0	90 (24.33)	48.4, 150.8	<0.01
Carbohydrates (g/d)	243 (63.4)	125.9, 390.6	220 (39.4)	128.4, 311.0	280.2 (76.7)	125.9, 390.6	<0.01

Abbreviations: ns.—not significant (*p* > 0.05), BMI—body mass index, WC—waist circumference, FFM—fat-free mass, FM—fat mass, VAT—visceral adipose tissue, SAT—subcutaneous adipose tissue, TBW—total body water, ECW—extracellular water, ICW—intracellular water, BCM—body cell mass, ECM—extracellular mass, HOMA-IR—homeostatic model assessment insulin resistance, TC—total cholesterol, TG—triglycerides, HDL-C—high-density lipoprotein cholesterol, LDL-C—low-density lipoprotein cholesterol, CRP—c-reactive protein, VO_2_—volume oxygen, VCO_2_—volume carbon dioxide, RQ—respiratory quotient, REE—resting energy expenditure, MPA—moderate physical activity, VPA—vigorous physical activity, MVPA—moderate and vigorous physical activity, TST—total sleep time.

**Table 2:** Influence of anthropometric and body composition components on REE.

Basic Parameters	*p*-Value	β ± SE	95%Cl	Adj. R^2^
Age (years)	ns.	-	-	-
Sex	<0.0001	535.7 ± 65.6	405.0, 666.2	0.454
Body weight (kg)	<0.0001	19.8 ± 2.4	15.6, 24.1	0.523
Height (cm)	<0.0001	26.8 ± 3.4	20.0, 33.7	0.427
BMI (kg/m^2^)	<0.0001	68.6 ± 11.9	45.0, 92.3	0.421
WC (cm)	<0.0001	22.1 ± 2.9	16.4, 27.8	0.426
**Body composition parameters**				
FFM (kg)	<0.0001	28.8 ± 2.8	23.2, 34.3	0.572
FM (kg)	<0.001	25.5 ± 6.1	13.3, 37.8	0.171
VAT (cm^2^)	<0.0001	3.0 ± 1.2	1.6, 4.5	0.266
SAT (cm^2^)	<0.05	3.5 ± 1.3	0.9, 6.1	0.064
VAT/SAT	<0.0001	366.3 ± 65.6	239.7, 492.9	0.244
TBW (Lt)	<0.0001	37.2 ± 3.7	29.8, 44.6	0.565
ECW (Lt)	<0.0001	100.7 ± 12.0	76.7, 124.7	0.466
ICW (Lt)	<0.0001	38.7 ± 5.6	27.6, 49,7	0.375
BCM (kg)	<0.0001	47.4 ± 4.6	38.2, 56.6	0.570
ECM (kg)	<0.0001	66.9 ± 7.0	53.0, 80,8	0.535
Protein mass (kg)	<0.0001	106.7 ± 13.7	79.3, 134.1	0.429
Muscle mass (kg)	<0.0001	44.8 ± 4.5	35.8, 53.7	0.554
Dry weight (kg)	<0.0001	19.1 ± 2.1	15.0, 23.3	0.516

Abbreviations: ns.—not significant (*p* > 0.05), REE—resting energy expenditure, Adj. R^2^—the adjusted coefficient of determination, β ± SE—regression coefficient ± standard error, Cl—confidence interval, BMI—body mass index, WC—waist circumference, FFM—fat-free mass, FM—fat mass, VAT—visceral adipose tissue, SAT—subcutaneous adipose tissue, TBW—total body water, ECW—extracellular water, ICW—intracellular water, BCM—body cell mass, ECM—extracellular mass.

**Table 3:** Influence of biochemical parameters on REE–crude model and model adjusted for sex and FFM (kg).

	Crude Model				Model 1 Adjusted for Sex, FFM			
Biochemical Parameters	*p*-Value	β ± SE	95%Cl	Adj. R^2^	*p*-Value	β ± SE	95%Cl	Adj. R^2^
TC (mg/dL)	ns.	-	-	-	ns.	-	-	-
HDL-C (mg/dL)	<0.05	−6.6 ± 3.1	−13.0, −0.4	0.045	<0.05	4.8 ± 2.3	0.1, 9.4	0.590
LDL-C (mg/dL)	ns.	-	-	-	ns.	-	-	-
TG (mg/dL)	<0.001	3.3 ± 0.9	1.5, 5.0	0.144	ns.	-	-	-
CRP (mg/L)	ns.	-	-	-	ns.	-	-	-
Fasting blood glucose (mg/dL)	ns.	-	-	-	ns.	-	-	-
Fasting insulin (μU/mL)	<0.05	22.1 ± 9.6	3.0, 41.3	0.054	ns.	-	-	-
HOMA-IR	<0.05	83.1 ± 36.1	11.1, 155.1	0.055	ns.	-	-	-

Abbreviations: ns.—not significant (*p* > 0.05), REE—resting energy expenditure, Adj. R^2^—the adjusted coefficient of determination, β ± SE—regression coefficient ± standard error, Cl—confidence interval, HOMA-IR—homeostatic model assessment insulin resistance, TC—total cholesterol, TG—triglycerides, HDL-C—high-density lipoprotein cholesterol, LDL-C—low-density lipoprotein cholesterol, CRP—c-reactive protein.

**Table 4:** Influence of lifestyle factors on REE–crude model and model adjusted for sex and FFM (kg).

	Crude Model				Model 1 Adjusted for Sex, FFM			
Physical Activity and Sleep Parameters	*p*-Value	β ± SE	95%Cl	Adj. R^2^	*p*-Value	β ± SE	95%Cl	Adj. R^2^
MPA (min/day)	<0.0001	5.8 ± 1.3	3.2, 8.4	0.201	<0.05	2.1 ± 1.0	0.3, 4.2	0.589
VPA (min/day)	<0.0001	11.4 ± 2.7	6.0, 16.7	0.185	ns.	-	-	-
MVPA (min/day)	<0.0001	4.3 ± 0.9	2.5, 6.1	0.221	<0.05	1.5 ± 0.8	0.1, 3.0	0.589
TST (min/night)	ns.	-	-	-	ns.	-	-	-
**Diet parameters**								
Energy (kcal/d)	<0.01	0.7 ± 0.2	0.3, 1.2	0.218	ns. (0.06)			
Protein (g/d)	ns.	-	-	-	ns.	-	-	-
Fats (g/d)	ns.	-	-	-	ns.	-	-	-
Carbohydrates (g/d)	ns.	-	-	-	ns.	-	-	-

Abbreviations: ns.—not significant (*p* > 0.05), REE—resting energy expenditure, Adj. R^2^—the adjusted coefficient of determination, β ± SE—regression coefficient ± standard error, Cl—confidence interval, MPA—moderate physical activity, VPA—vigorous physical activity, MVPA—moderate and vigorous physical activity, TST—total sleep time.

**Table 5:** Independent contributions of MPA, MVPA, and HDL-C to REE beyond FFM (kg) and sex: regression and partial correlation results.

Model	Adj. R^2^	ΔR^2^ (Increase)	*p*-Value (ΔR^2^)	Partial r	*p*-Value (Partial r)
FFM + sex	0.570	0.00	-	-	-
FFM + sex + MPA	0.589	0.02	<0.05	0.233	<0.05
FFM + sex + MVPA	0.589	0.02	<0.05	0.232	<0.05
FFM + sex + HDL-C	0.590	0.02	<0.05	0.236	<0.05

Abbreviations: REE—resting energy expenditure, Adj. R^2^—the adjusted coefficient of determination, MPA—moderate physical activity, MVPA—moderate and vigorous physical activity, HDL-C—high-density lipoprotein cholesterol, ΔR^2^—change in adjusted R^2^, Partial r—partial correlation coefficient.

## Data Availability

Data presented in this study are available on request from the corresponding author.
